# Supplementary material for: High variability in transmission of SARS-CoV-2 within households and implications for control
Source: PLoS One. 2021 Nov 10;16(11):e0259097. doi: 10.1371/journal.pone.0259097 (PMC8580228; doi:10.1371/journal.pone.0259097)
Supplement: S1 File — (DOCX) [file pone.0259097.s001.docx]

**Supplementary material for “High variability in transmission of SARS-CoV-2 within households and implications for control,” authored by Damon J.A. Toth et al.**

**Supplemental Methods**

***Survey data***

Answers to the following questions for individual household members were used in our analysis:

1. “Have you ever been tested for coronavirus (also called SARS-CoV-2 or COVID-19)?” (Yes or No)

If the answer to 1) was Yes, the following two questions were asked:

1. What was the result? (Positive; Negative; Have not received test result; or Don’t know)
2. When were you tested? (MM/DD/YYYY)

All individuals who answered “Yes” to question 1 and “Positive” to question 2 were classified as “reported a prior positive test,” and all other surveyed individuals were classified as “did not report a prior positive test,” as described in the main text. For individuals who reported a prior positive test and also received an antibody test, we used the answer to question 3, compared to the collection date of serology, to construct Table S2.

***Alternate model with variability in household importation***

For the alternate model, the formula for $M_{kn}$ for a given household size $n\geq2$ becomes

$$M_{kn}\left( p_{c},d_{c},p_{h},d_{h} \right)=\left\{ \begin{matrix} F_{0n}\left( p_{c},d_{c} \right), & k=0 \\ \sum_{i=1}^{k} F_{in}\left( p_{c},d_{c} \right)T_{i,k-i,n-i}\left( p_{h},d_{h} \right), & k=1,\ldots,n-1 \\ 1-\sum_{k=0}^{n-1} M_{kn}\left( p_{c},d_{c},p_{h},d_{h} \right), & k=n \end{matrix} \right.$$

For households of size $n=$ 1, $M_{01}\left( p_{c},d_{c} \right)=F_{01}\left( p_{c},d_{c} \right)$ and $M_{11}\left( p_{c},d_{c}, \right)=1-F_{01}\left( p_{c},d_{c} \right)$. The function $F_{yz}\left( p,d \right)$ is defined in the main text (probability mass function of the beta-binomial distribution with boundary case definitions at $d=0$ and $d\to\infty$), where in this case $y$ is the number of community acquisitions and $z$ is the total number of household members. The main-text model is a special case of this alternate model, with $d_{c}\to\infty$.

The likelihood equation is the same as in the main text, but with the additional element $d_{c}$ in the vector $\boldsymbol{\theta}$ of variables to be optimized:

$$\boldsymbol{\theta}=\left( p_{c},d_{c},p_{h},d_{h},\phi_{V},\phi_{A},\pi_{V},\pi_{A} \right)$$

and $M_{kn}\left( p_{c},p_{h},d_{h} \right)$ in the likelihood equation is replaced with $M_{kn}\left( p_{c},d_{c},p_{h},d_{h} \right)$ as defined above.

We found the MLE and single-parameter confidence intervals using the same procedure described in the main text, and further assessed uncertainty of $d_{h}$ by solving for the MLE of the other 7 variables when fixing it at its boundary values 0 and ∞. We also compared the likelihood at the MLE of the alternate model to that of the main text model using the likelihood ratio test, to determine whether the main text model result could be rejected in favor of the alternate model by this criterion. As an additional comparison, we used the Bayesian information criterion to score the alternate model against the main text model, using 9224 as the number of data points (number of households) and 7 and 8 as the number of parameters for the main-text model and alternate model, respectively.

***Beta-binomial distribution at limits of dispersion parameter:*** $\boldsymbol{d\to\infty}$ ***and*** $\boldsymbol{d\to0}$

Our likelihood equations make use of the beta-binomial probability distribution, parameterized with an average probability $p$ and a dispersion parameter $d$. The probability mass function $F$ for positive, finite values of $d$ is

$$F_{yz}\left( p,d \right)=\left( \begin{matrix} z \\ y \end{matrix} \right)\frac{B\left( y+dp,z-y+d\left( 1-p \right) \right)}{B\left( dp,d\left( 1-p \right) \right)}, y=0,1,\ldots,z$$

We use $F_{yz}\left( p,d \right)$ to quantify the distribution of household transmissions directly from a single infected household member, where$y$ is the number of transmissions, $z$ is the number of susceptible household members, $p=p_{h}$, and $d=d_{h}$. In our alternate model we also use $F_{yz}\left( p,d \right)$ to quantify the distribution of community acquisitions among members of a household from non-household members, where $y$ is the number of community acquisitions, $z$ is the total number of household members, $p=p_{c}$, and $d=d_{c}$.

Here, we derive the formula for $F_{yz}\left( p,d \right)$ at the boundaries of the range of possible values for $d$: $d\to\infty$ and $d\to0$. To do this, we rewrite $F_{yz}\left( p,d \right)$ in an alternate form. First, using the property $B\left( x,y \right)={\Gamma\left( x \right)\Gamma\left( y \right)}/{\Gamma\left( x+y \right)}$:

$$F_{yz}\left( p,d \right)=\left( \begin{matrix} z \\ y \end{matrix} \right)\frac{\Gamma\left( d \right)}{\Gamma\left( z+d \right)}\frac{\Gamma\left( y+dp \right)}{\Gamma\left( dp \right)}\frac{\Gamma\left( z-y+d\left( 1-p \right) \right)}{\Gamma\left( d\left( 1-p \right) \right)}$$

Then using the property, for positive integer $n$, $\Gamma\left( z+n \right)=z\left( z+1 \right)\cdots\left( z+n-1 \right)\Gamma\left( z \right)$:

$$F_{yz}\left( p,d \right)=\left\{ \left( \begin{matrix} z \\ y \end{matrix} \right)\begin{matrix} \frac{d\left( 1-p \right)\left( d\left( 1-p \right)+1 \right)\cdots\left( d\left( 1-p \right)+z-1 \right)}{d\left( d+1 \right)\cdots\left( d+z-1 \right)} & y=0 \\ \frac{\left[ dp\left( dp+1 \right)\cdots\left( dp+y-1 \right) \right]\left[ d\left( 1-p \right)\left( d\left( 1-p \right)+1 \right)\cdots\left( d\left( 1-p \right)+z-y-1 \right) \right]}{d\left( d+1 \right)\cdots\left( d+z-1 \right)} & 0<y<z \\ \frac{dp\left( dp+1 \right)\cdots\left( dp+z-1 \right)}{d\left( d+1 \right)\cdots\left( d+z-1 \right)} & y=z \end{matrix} \right.$$

In the following we rewrite the numerators and denominators in powers of $d$. Only the lowest and highest powers of $d$ will matter for taking the limits that follow, so the other terms within “$\cdots$” are not shown.

$$F_{yz}\left( p,d \right)=\left\{ \left( \begin{matrix} z \\ y \end{matrix} \right)\begin{matrix} \frac{\left( 1-p \right)\left( z-1 \right)!d+\cdots+\left( 1-p \right)^{z}d^{z}}{\left( z-1 \right)!d+\cdots+d^{z}} & y=0 \\ \frac{p\left( 1-p \right)\left( y-1 \right)!\left( z-y-1 \right)!d^{2}+\cdots+p^{y}\left( 1-p \right)^{z-y}d^{z}}{\left( z-1 \right)!d+\cdots+d^{z}} & 0<y<z \\ \frac{p\left( z-1 \right)!d+\cdots+p^{z}d^{z}}{\left( z-1 \right)!d+\cdots+d^{z}} & y=z \end{matrix} \right.$$

When taking the limit $d\to\infty$, we note that each fraction has highest order $d^{z}$ in both numerator and denominator, so the limit will be the ratio of the coefficients of that term:

$$\lim_{d\to\infty}\left( \begin{matrix} z \\ y \end{matrix} \right)\frac{B\left( y+dp,z-y+d\left( 1-p \right) \right)}{B\left( dp,d\left( 1-p \right) \right)} =\left( \begin{matrix} z \\ y \end{matrix} \right)p^{y}\left( 1-p \right)^{z-y}$$

When taking the limit $d\to0$, we note that the fraction for cases $y=0$ and $y=z$ have lowest order term $d$ in both the numerator and denominator, so the limit will be the ratio of the coefficients on those terms. The fraction for the $0<y<z$ case has only powers of $d^{2}$ and higher in the numerator, and a nonzero $d$ term in the denominator, so the limit is 0:

$$\lim_{d\to0}\left( \begin{matrix} z \\ y \end{matrix} \right)\frac{B\left( y+dp,z-y+d\left( 1-p \right) \right)}{B\left( dp,d\left( 1-p \right) \right)} =\left\{ \begin{matrix} 1-p, & y=0 \\ 0, & 0<y<z \\ p, & y=z \end{matrix} \right.$$

***Direct transmission probabilities*** $\boldsymbol{H}_{\boldsymbol{xyz}}$

Here we derive the formulae for $H_{xyz}$: the probability of $y$ transmissions to $z$ susceptible household members directly from $x$ infected members

As described in the main text, we first define the probabilities for transmissions directly from $x=1$ infected household member:

$$H_{1yz}=F_{yz}\left( p_{h},d_{h} \right)=\left\{ \begin{matrix} \left\{ \begin{matrix} 1-p_{h}, & y=0 \\ 0, & 0<y<z \\ p_{h}, & y=z \end{matrix} \right., & d_{h}=0 \\ \left( \begin{matrix} z \\ y \end{matrix} \right)\frac{B\left( y+d_{h}p_{h},z-y+d_{h}\left( 1-p_{h} \right) \right)}{B\left( d_{h}p_{h},d_{h}\left( 1-p_{h} \right) \right)}, & 0<d_{h}<\infty\\ \left( \begin{matrix} z \\ y \end{matrix} \right){p_{h}}^{y}\left( 1-p_{h} \right)^{z-y}, & d_{h}=\infty\end{matrix} \right.$$

Next consider $x=2$ infected household members. The probability that $y=0$ transmissions occur is the probability that both infected members transmit to 0 others: $H_{20z}=H_{10z}H_{10z}$. To calculate the probability that $y>0$ transmissions occur from the two infected members, it is convenient to consider the two infected individuals having transmission opportunities in sequence, say A followed by B. If A transmits to any household members, this reduces the number of susceptible members remaining for B to infect. For example, the probability that $y=1$ is the probability that A transmits to 0 of $z$ and B transmits to 1 of $z$, plus the probability that A transmits to 1 of $z$ and B transmits to 0 of $z-1$ remaining susceptible members: $H_{21z}=H_{10z}H_{11z}+H_{11z}H_{1,0,z-1}$. The calculation follows a similar pattern for $y=2$: $H_{22z}=H_{10z}H_{12z}+H_{11z}H_{1,1,z-1}+H_{12z}H_{1,0,z-2}$. It follows that:

$$H_{2yz}=\sum_{i=0}^{y} H_{1iz}H_{1,y-i,z-i}$$

Now for $x=3$ infected household members, we can use the fact that we have already calculated $H_{2yz}$, which covers the transmission probabilities from two of the three infected members, and then we include the probability that third member transmits to any remaining susceptible members that the first two did not infect:

$$H_{3yz}=\sum_{i=0}^{y} H_{2iz}H_{1,y-i,z-i}$$

Following this pattern, we continue calculating $H_{xyz}$ for each $x$ in increasing sequence:

$$H_{xyz}=\sum_{i=0}^{y} H_{x-1,i,z}H_{1,y-i,z-i}$$

***Total transmission probabilities*** $\boldsymbol{T}_{\boldsymbol{xyz}}$

Here we derive the formulae for $T_{xyz}$: the probability of $y$ total transmissions to $z$ initially susceptible members from $x$ initially infected members. In other words, $T_{xyz}$ is the probability that the final household outbreak size is $x+y$, given that $x$ household members were initially infected and $z$ household members were initially susceptible.

First, we note that $T_{x0z}=H_{x0z}$ for all possible ($x,z$) pairs, because if the initial $x$ infected members do not transmit to anyone ($y=$ 0), the household outbreak is over and the final size has been reached. Next we consider the probability of $y=$ 1 total transmissions, which occurs when the initial $x$ infected members transmit directly to 1 other (with probability $H_{x1z}$), who then does not subsequently transmit to any of the remaining $z-1$ susceptible members (with probability $H_{1,0, z-1}=T_{1,0, z-1}$). Hence,

$$T_{x1z}=H_{x1z}T_{1,0, z-1}$$

For $y=$ 2 total transmissions, we must include the probability that the initial $x$ infected members transmit directly to 2 others who then transmit to none and the probability that the initial $x$ infected members transmit directly to 1 other who then produces an outbreak among the remaining susceptible members with 1 total transmission:

$$T_{x2z}=H_{x2z}T_{2,0, z-2}+H_{x1z}T_{1,1, z-1}$$

Following similar logic for $y=$ 3, and making use of the $T_{x0z}$, $T_{x1z}$, and $T_{x2z}$ values already calculated, we arrive at:

$$T_{x3z}=H_{x3z}T_{3,0, z-3}+H_{x2z}T_{2,1, z-2}+H_{x1z}T_{1,2, z-1}$$

The general formula calculated for increasing values of $y$ is:

$$T_{xyz}=\sum_{i=0}^{y-1} H_{x,y-i,z}T_{y-i,i, z-y+i}$$

***Within-household reproduction numbers and threshold condition***

We define the within-household reproduction number $R_{h}$ as the expected number of household transmissions directly from an infected person who acquired infection in the community and has no non-susceptible housemates. Let $h_{i}$ be the fraction of households with size $i$, up to a maximum size $N$. Then the mean $\mu$ and variance $\sigma^{2}$ of the household size distribution are

$$\mu=\sum_{i=1}^{N} \left( ih_{i} \right), \sigma^{2}=\sum_{i=1}^{N} \left( i^{2}h_{i} \right)-\mu^{2}$$

Let $c_{i}$ be the probability that a randomly chosen person has $i$ housemates. The probability that a randomly chosen person lives in a house of *total* size $i$ (including themselves) is ${ih_{i}}/\mu$, so

$$c_{i}={\left( i+1 \right)h_{i+1}}/\mu$$

Then $R_{h}$ is $p_{h}$ times the mean number of housemates of a randomly chosen person:

$$R_{h}=p_{h}\sum_{i=1}^{N-1} \left( ic_{i} \right)=p_{h}\left( \sum_{i=1}^{N} \left( i^{2}h_{i} \right)-\sum_{i=1}^{N} \left( ih_{i} \right) \right)/\mu=p_{h}\left( \mu+{\sigma^{2}}/\mu-1 \right)$$

We also define an alternate within-household reproduction number $R_{h}^{*}$ as the expected total number of transmissions in the household of an infected person who acquired infection in the community and has no initially non-susceptible housemates. Given that a person acquiring infection in the community has $i$ susceptible housemates, the probability that $j$ of their housemates become infected before the household outbreak terminates is $T_{1ji}\left( p_{h},d_{h} \right)$, as defined in Section 2.2 of the main text. The expected total number of transmissions in their household will be then be $\sum_{j=1}^{i} jT_{1ji}\left( p_{h},d_{h} \right)$. So, the household reproduction number formula is:

$$R_{h}^{*}=\sum_{i=1}^{N-1} c_{i}\sum_{j=1}^{i} jT_{1ji}\left( p_{h},d_{h} \right)=\sum_{i=1}^{N-1} \frac{\left( i+1 \right)h_{i+1}}{\mu}\sum_{j=1}^{i} jT_{1ji}\left( p_{h},d_{h} \right)$$

For the high-variability boundary case at $d_{h}=0$, we have that

$$\sum_{j=1}^{i} jT_{1ji}\left( p_{h},0 \right)=ip_{h}$$

because $T_{1ji}\left( p_{h},0 \right)=p_{h}$ when $j=i$ and 0 for other nonzero values of $j$ (reflecting all-or-nothing transmission). It follows that $R_{h}=R_{h}^{*}$ when $d_{h}=0$. This makes intuitive sense because in the all-or-nothing scenario, when the index person transmits, all in the household are infected directly, and there is no one left to infect in subsequent generations, so the final household outbreak size is entirely reflected in $R_{h}$.

We next investigate the implications of our $R_{h}^{*}$ estimate for population-wide transmission control. The threshold condition delineating subcritical and supercritical transmission in the population occurs when the maximal eigenvalue of the matrix

$$\left[ \begin{matrix} R_{c} & R_{h}^{*} \\ R_{c} & 0 \end{matrix} \right]$$

exceeds one [30]. Here, $R_{c}$ is defined as the average number of community transmissions per infected individual (i.e., average number of transmissions to people not in the infected individual’s household). The occurrence of $R_{c}$ in both rows of the matrix reflects an assumption that its value applies to the transmissibility of people who acquire their own infection in the community and in their household. The zero element in the lower-right corner of the matrix reflects the fact that the $R_{h}^{*}$ people on average who acquire infection in their household do not transmit further in their household, by definition, because $R_{h}^{*}$ was derived from the final household outbreak size equations encompassed in $T_{xyz}$.

The maximal eigenvalue exceeding one produces the following threshold condition:

$$\frac{R_{c}}{2}\left( 1+\sqrt{1+4{R_{h}^{*}}/{R_{c}}} \right)>1$$

This is equivalent to

$$R_{c}\left( R_{h}^{*}+1 \right)>1$$

If the threshold condition is met and $R_{h}^{*}$ is fixed, then the system can be pushed below threshold by reducing $R_{c}$ such that

$$R_{c}<\frac{1}{R_{h}^{*}+1}$$

If $R_{c}$ is fixed and less than one, then the system can be pushed below threshold by reducing $R_{h}^{*}$ such that

$$R_{h}^{*}<\frac{1}{R_{c}}-1$$

***Relationship between beta distributed probability and dynamic transmission parameters***

If an infected person’s duration of infectiousness is $\tau$ and the transmission rate to a contact is $\beta$, the probability that transmission to the contact occurs is $p=1-e^{-\beta\tau}$. We assume $\tau$ is fixed and $\beta$ is a gamma distributed random variable with shape $k$ and rate $r$. Then, the first and second moments of the random variable $p$ are:

$$E\left[ p \right]=\int_{0}^{\infty} \frac{r^{k}}{\Gamma\left( k \right)}x^{k-1}e^{-rx}\left( 1-e^{-\tau x} \right)dx=1-\left( \frac{r}{r+\tau} \right)^{k}$$

$$E\left[ p^{2} \right]=\int_{0}^{\infty} \frac{r^{k}}{\Gamma\left( k \right)}x^{k-1}e^{-rx}\left( 1-e^{-\tau x} \right)^{2}dx=1-2\left( \frac{r}{r+\tau} \right)^{k}+\left( \frac{r}{r+2\tau} \right)^{k}$$

The variance is then

$$\mathrm{Var}\left[ p \right]=E\left[ p^{2} \right]-\left( E\left[ p \right] \right)^{2}=\left( \frac{r}{r+2\tau} \right)^{k}-\left( \frac{r}{r+\tau} \right)^{2k}$$

We then equate the mean and variance to those of the beta distribution with mean $p_{h}$ and dispersion $d_{h}$, which we used in our MLE model in the main text.

$$p_{h}=1-\left( \frac{r}{r+\tau} \right)^{k}$$

$$\frac{p_{h}\left( 1-p_{h} \right)}{d_{h}+1}=\left( \frac{r}{r+2\tau} \right)^{k}-\left( \frac{r}{r+\tau} \right)^{2k}$$

Combining those two equations yields

$$\left( \left( 1-p_{h} \right)d_{h}+1 \right)^{1/k}\left( 2-\left( 1-p_{h} \right)^{1/k} \right)-\left( d_{h}+1 \right)^{1/k}=0$$

$$r=\frac{\tau\left( 1-p_{h} \right)^{1/k}}{1-\left( 1-p_{h} \right)^{1/k}}$$

We solved the first equation for $k$, which is independent of the assumption for $\tau$, using our MLE estimates of $p_{h}$ and $d_{h}$. We applied each of the ($p_{h}$,$d_{h}$) pairs from our parametric bootstrap analysis to this equation to derive the confidence interval for $k$.

**Supplemental Results**

The alternate model produced an estimate for the new dispersion parameter $d_{c}=$ 2.1 (0.89 – 7.5) and altered estimates for the other 7 parameters compared to their values for the main text model (Table S4). The log likelihood at this MLE was about 1 greater than the log likelihood produced by the main text result (Table S5), suggesting that the main text model (equivalent to the alternate model with $d_{c}=\infty$) cannot be rejected with high confidence in favor of the alternate model by the likelihood ratio test (P = 0.14). The Akaike information criterion for the alternate model is 2361.50 compared to 2361.68 for the main text ($d_{c}=\infty$) model, a difference too small to conclude favorability of one model over the other.

The conclusion of high household transmission variability from the main-text model is consistent under this alternate model, with the MLE occurring at low value of the dispersion parameter $d_{h}=$ 0.21, and the low-variability binomial model $d_{h}=$ $\infty$ can be rejected with P = 0.024. However, uncertainty ranges become wider at higher levels of overdispersion in household risk of community acquisition. This is illustrated by the fact that a model assuming no household transmission ($p_{h}=0$), i.e., all household cases explained by acquisitions outside the households with high overdispersion ($d_{c}=$ 0.5), cannot be rejected with very high confidence (P = 0.079). Thus, the alternate explanation for the distribution of household cases may not be definitively ruled out by our data (Table S5).

Table S1. Fraction of household data from each county in the state of Utah

|  | Data | Utah population |
| --- | --- | --- |
| Cache County | 11.1% | 4.0% |
| Davis County | 11.4% | 11.2% |
| Salt Lake County | 41.5% | 36.6% |
| Summit County | 3.1% | 1.3% |
| Utah County | 12.5% | 19.6% |
| Washington County | 11.4% | 5.4% |
| Weber County | 8.9% | 8.1% |
| Other 22 Counties | 0.0% | 13.9% |

Data refers to the fraction of households that contributed serology data for antibody testing; Utah population refers to the fraction of the overall state population that resides in each county (US Census data).

Table S2. Intervals between reported prior positive test and antibody test results

| Interval range | Number | Number antibody positive |
| --- | --- | --- |
| 1 – 7 days | 3 | 0 |
| 8 – 14 days | 11 | 9 |
| 15 – 21 days | 10 | 9 |
| 22 – 28 days | 10 | 9 |
| 29 – 35 days | 8 | 6 |
| 36 – 42 days | 10 | 6 |
| 43 – 56 days | 10 | 9 |
| 57 – 70 days | 7 | 6 |
| 71 – 129 days | 6 | 4 |

Number in middle column is the count of individuals whose reported date of a prior positive test occurred within the given range of days before the date of antibody testing, and the third column gives the number of those who tested positive for antibodies.

Table S3. Demographic distributions of surveyed individuals

|  | Data | Utah | U.S. |
| --- | --- | --- | --- |
| **Age and Sex** |  |  |  |
| Age 0 to 4 years | 1.6% | 7.7% | 6.1% |
| Age 5 to 14 years | 6.6% | 16.4% | 12.6% |
| Age 15 to 24 years | 13.2% | 16.2% | 13.0% |
| Age 25 to 34 years | 15.8% | 14.7% | 14.0% |
| Age 35 to 44 years | 16.6% | 13.8% | 12.6% |
| Age 45 to 54 years | 12.5% | 10.2% | 12.6% |
| Age 55 to 64 years | 13.8% | 9.5% | 12.8% |
| Age 65 to 74 years | 13.0% | 6.8% | 9.8% |
| Age 75 to 84 years | 5.7% | 3.4% | 4.8% |
| Age 85 years and over | 1.2% | 1.2% | 1.8% |
| Female persons | 52.0% | 49.6% | 50.8% |
| **Race and Hispanic Origin** |  |  |  |
| White alone | 93.8% | 90.6% | 76.3% |
| Black or African American alone | 0.7% | 1.5% | 13.4% |
| American Indian and Alaska Native alone | 0.6% | 1.6% | 1.3% |
| Asian alone | 2.3% | 2.7% | 5.9% |
| Native Hawaiian and Other Pacific Islander alone | 0.5% | 1.1% | 0.2% |
| Two or More Races | 2.0% | 2.6% | 2.8% |
| Hispanic or Latino | 8.5% | 14.4% | 18.5% |
| White alone, not Hispanic or Latino | 87.9% | 77.8% | 60.1% |
| **Education** |  |  |  |
| High school graduate or higher, persons age 25+ | 98.2% | 92.3% | 88.0% |
| Bachelor’s degree or higher, persons age 25+ | 57.1% | 34.0% | 32.1% |

Data refers to the fraction of surveyed individuals who reported each characteristic in our survey results. Utah and U.S. columns contain data from the US Census (Vintage 2019 Population Estimates Program).

Table S4. Alternate model results: allowing variability in importation probability by household

| Value | MLE estimate (95% interval) |
| --- | --- |
| Mean community acquisition probability ($p_{c})$ | 0.56% (0.44% – 0.70%) |
| Household community acquisition dispersion ($d_{c}$) | 2.1 (0.89 – 7.5) |
| Mean per-capita household transmission probability ($p_{h}$) | 27% (16% – 41%) |
| Per-capita household transmission dispersion ($d_{h}$) | 0.21 (0 – 3.4) |
| Probability that surveyed, infected person reported a prior positive test ($\phi_{V}$) | 72% (62% – 82%) |
| Probability that antibody test of person with prior infection was positive ($\phi_{A}$) | 87% (77% – 94%) |
| Probability that surveyed, uninfected person did not report a prior positive test ($\pi_{V}$) | 99.92% (99.86% – 99.97%) |
| Probability that antibody test of person with no prior infection was negative ($\pi_{A}$) | 99.3% (99.2% – 99.5%) |

Confidence intervals for MLE derived from the likelihood ratio test, varying each individual parameter while fixing other parameters at their MLE values.

Table S5. Comparison of alternate model MLE to main-text and other models

| Fixed values | $\hat{p}_{c}$ | $\hat{d}_{c}$ | $\hat{p}_{h}$ | $\hat{d}_{h}$ | $\hat{\phi}_{V}$ | $\hat{\phi}_{A}$ | $\hat{\pi}_{V}$ | $\hat{\pi}_{A}$ | Log likelihood | # of optimized parameters | Rejection P value |
| --- | --- | --- | --- | --- | --- | --- | --- | --- | --- | --- | --- |
| None | 0.56% | 2.1 | 27% | 0.21 | 72% | 87% | 99.92% | 99.3% | −1172.75 | 8 | - |
| $d_{c}=$ ∞ | 0.41% | **∞*** | 36% | 0.43 | 72% | 86% | 99.94% | 99.3% | −1173.84 | 7 | 0.14 |
| $d_{h}=$ 0 | 0.60% | 1.7 | 24% | **0*** | 72% | 87% | 99.93% | 99.3% | −1172.86 | 7 | 0.64 |
| $d_{h}=$ ∞ | 0.77% | 0.5 | 0% | **∞*** | 72% | 88% | 99.92% | 99.3% | −1175.29 | 7 | 0.024 |
| $p_{h}=$ 0% | 0.77% | 0.5 | **0%*** | N/A | 72% | 88% | 99.92% | 99.3% | −1175.29 | 6 | 0.079 |

*Values that were fixed for the model in that row; other relevant values were optimized by MLE. P values were derived from the likelihood ratio test, compared to the likelihood of the overall MLE in the top row: twice the difference in log likelihood compared to the chi-squared distribution with degrees of freedom equal to the difference in the number of optimized parameters. The model with household transmission probability $p_{h}$ fixed at 0% optimized only 6 parameters because the transmission dispersion parameter $d_{h}$ is irrelevant with no transmission, hence we used 2 degrees of freedom for the reference chi-squared distribution when comparing the likelihood to the full 8-parameter model. For the model with $d_{h}$ fixed at ∞ (binomial transmission model), the optimum occurred at the boundary $p_{h}$ = 0, producing the same likelihood as the adjacent model but different P-value as the reference chi-squared distribution has 1 degree of freedom.

Figure S1. Two-dimension confidence regions for $p_{c}$ paired with each other parameter


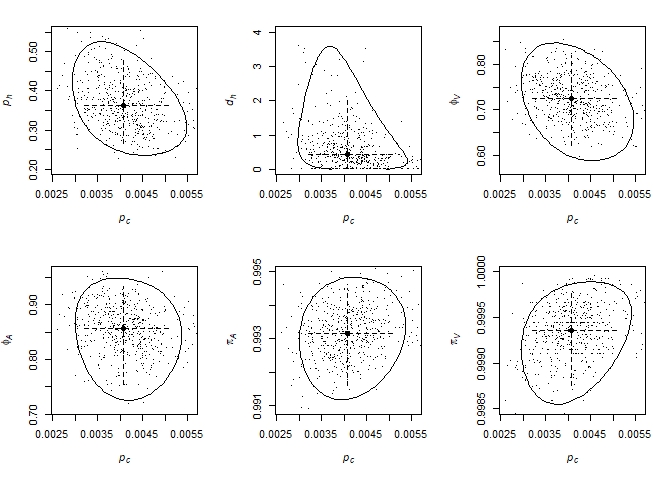


Solid curves are the 2-dimensional confidence regions derived from the likelihood ratio test, comparing the likelihood ratio statistic to the 95^th^ percentile of the chi-squared distribution with 2 degrees of freedom. Large solid circle is the MLE estimate and dashed lines are the confidence intervals for each individual parameter derived from the likelihood ratio test (Table 1 main text). Small dots are the MLE estimates from each of 500 simulated data sets generated using parameter values set at the MLE from the actual data (parametric bootstrap).

Figure S2. Two-dimension confidence regions for $p_{h}$ paired with each other parameter


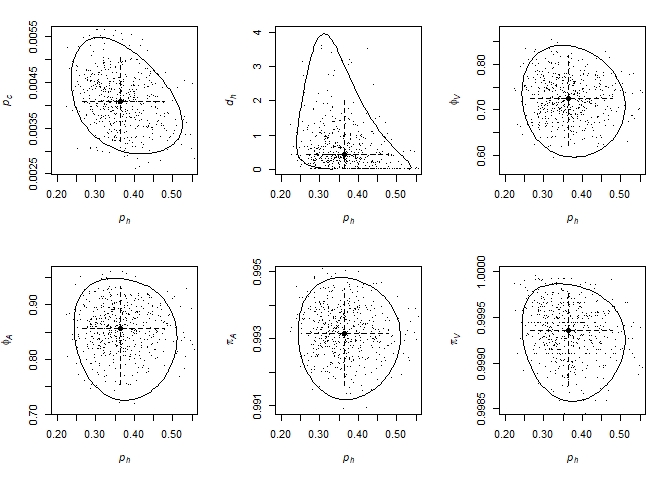


Solid curves are the 2-dimensional confidence regions derived from the likelihood ratio test, comparing the likelihood ratio statistic to the 95^th^ percentile of the chi-squared distribution with 2 degrees of freedom. Large solid circle is the MLE estimate and dashed lines are the confidence intervals for each individual parameter derived from the likelihood ratio test (Table 1 main text). Small dots are the MLE estimates from each of 500 simulated data sets generated using parameter values set at the MLE from the actual data (parametric bootstrap).

Figure S3. Two-dimension confidence regions for $d_{h}$ paired with each other parameter


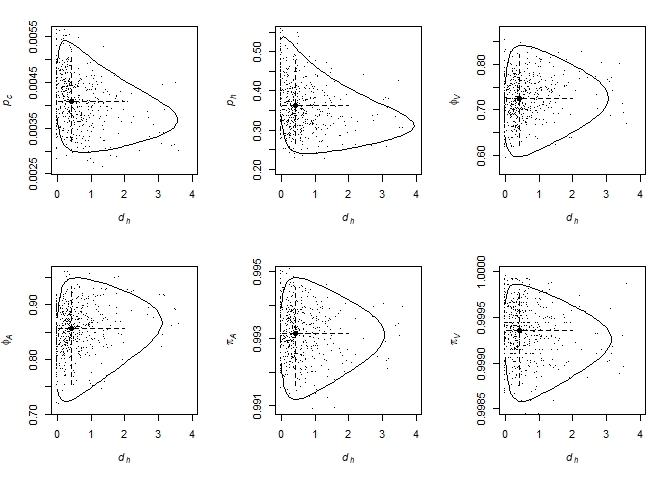


Solid curves are the 2-dimensional confidence regions derived from the likelihood ratio test, comparing the likelihood ratio statistic to the 95^th^ percentile of the chi-squared distribution with 2 degrees of freedom. Large solid circle is the MLE estimate and dashed lines are the confidence intervals for each individual parameter derived from the likelihood ratio test (Table 1 main text). Small dots are the MLE estimates from each of 500 simulated data sets generated using parameter values set at the MLE from the actual data (parametric bootstrap).

Figure S4. Two-dimension confidence regions for $\phi_{V}$ paired with each other parameter


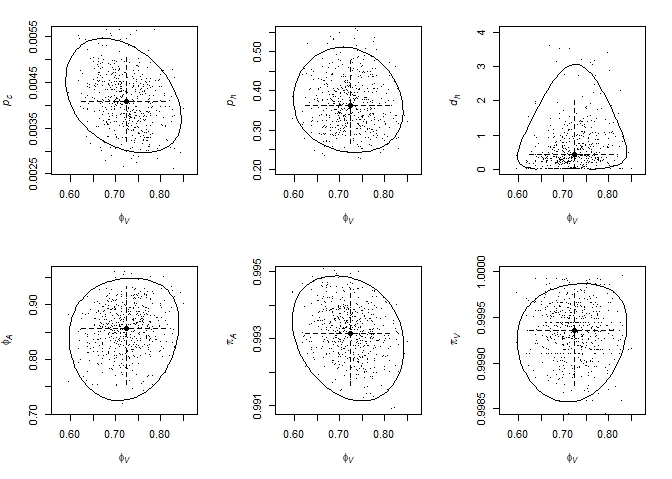


Solid curves are the 2-dimensional confidence regions derived from the likelihood ratio test, comparing the likelihood ratio statistic to the 95^th^ percentile of the chi-squared distribution with 2 degrees of freedom. Large solid circle is the MLE estimate and dashed lines are the confidence intervals for each individual parameter derived from the likelihood ratio test (Table 1 main text). Small dots are the MLE estimates from each of 500 simulated data sets generated using parameter values set at the MLE from the actual data (parametric bootstrap).

Figure S5. Two-dimension confidence regions for $\phi_{A}$ paired with each other parameter


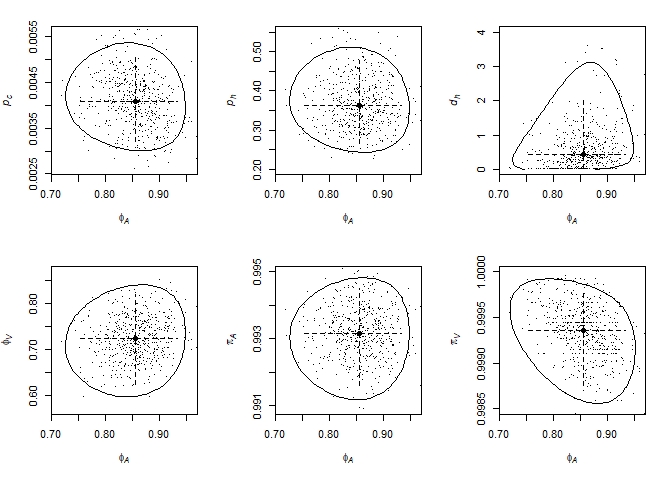


Solid curves are the 2-dimensional confidence regions derived from the likelihood ratio test, comparing the likelihood ratio statistic to the 95^th^ percentile of the chi-squared distribution with 2 degrees of freedom. Large solid circle is the MLE estimate and dashed lines are the confidence intervals for each individual parameter derived from the likelihood ratio test (Table 1 main text). Small dots are the MLE estimates from each of 500 simulated data sets generated using parameter values set at the MLE from the actual data (parametric bootstrap).

Figure S6. Two-dimension confidence regions for $\pi_{V}$ paired with each other parameter


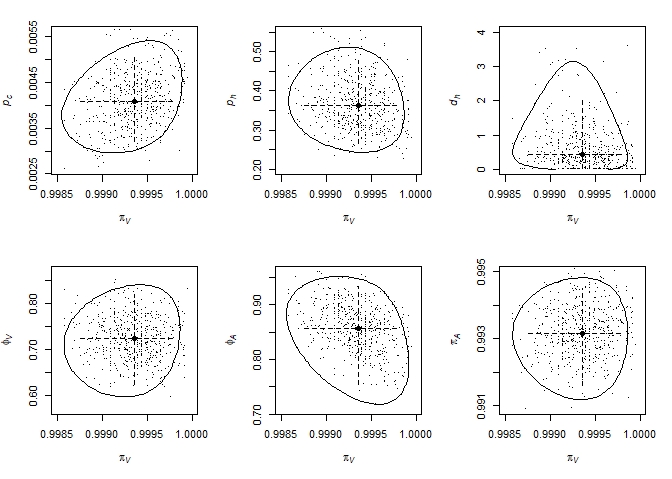


Solid curves are the 2-dimensional confidence regions derived from the likelihood ratio test, comparing the likelihood ratio statistic to the 95^th^ percentile of the chi-squared distribution with 2 degrees of freedom. Large solid circle is the MLE estimate and dashed lines are the confidence intervals for each individual parameter derived from the likelihood ratio test (Table 1 main text). Small dots are the MLE estimates from each of 500 simulated data sets generated using parameter values set at the MLE from the actual data (parametric bootstrap).

Figure S7. Two-dimension confidence regions for $\pi_{A}$ paired with each other parameter


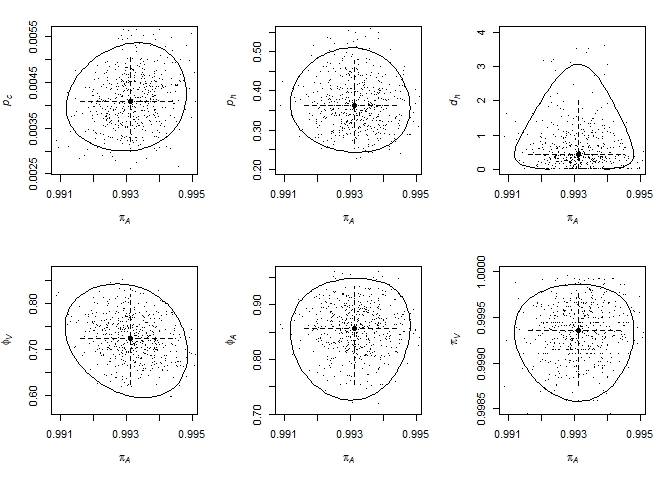


Solid curves are the 2-dimensional confidence regions derived from the likelihood ratio test, comparing the likelihood ratio statistic to the 95^th^ percentile of the chi-squared distribution with 2 degrees of freedom. Large solid circle is the MLE estimate and dashed lines are the confidence intervals for each individual parameter derived from the likelihood ratio test (Table 1 main text). Small dots are the MLE estimates from each of 500 simulated data sets generated using parameter values set at the MLE from the actual data (parametric bootstrap).
